# Development of MHFA-based 2-h educational program for early intervention in depression among office workers: A single-arm pilot trial

**DOI:** 10.1371/journal.pone.0208114

**Published:** 2018-12-07

**Authors:** Hiroaki Kubo, Hiromi Urata, Ryoko Katsuki, Miyako Hirashima, Shion Ueno, Yuriko Suzuki, Daisuke Fujisawa, Naoki Hashimoto, Keiji Kobara, Tetsuji Cho, Toshiko Mitsui, Shigenobu Kanba, Kotaro Otsuka, Takahiro A. Kato

**Affiliations:** 1 Department of Neuropsychiatry, Graduate School of Medical Sciences, Kyushu University, Fukuoka, Fukuoka, Japan; 2 TOTO Ltd., Kitakyushu, Fukuoka, Japan; 3 Department of Mental Health Policy, National Institute of Mental Health, National Center of Neurology and Psychiatry, Kodaira, Tokyo, Japan; 4 Department of Neuropsychiatry, Keio University School of Medicine, Shinjuku-ku, Tokyo, Japan; 5 Department of Psychiatry, Hokkaido University Graduate School of Medicine, Sapporo, Hokkaido, Japan; 6 Shimane Prefectural Counseling Center for Physical and Mental Health, Matsue, Shimane, Japan; 7 Mie Prefectural Mental Medical Center, Tsu, Mie, Japan; 8 Kitakyushu Mental Health Center, Kitakyushu, Fukuoka, Japan; 9 Department of Neuropsychiatry, School of Medicine, Iwate Medical University, Morioka, Iwate, Japan; Chiba Daigaku, JAPAN

## Abstract

**Objective:**

In the workplace depression and suicide are serious mental health problems. A lack of knowledge and mental health skills along with the stigma toward mental health problems often results in delays in seeking professional help. Interventions targeting not only persons with mental health problems but also people around the individual are warranted in order to encourage supporting behavior within entire workplace. In the present study, we investigated the efficacy of our newly developed educational training program in the management with depression and suicidal risk in the workplace as a single-arm pilot trial.

**Methods:**

The program is a two-hour (2-h) training course for employees based on the Mental Health First Aid (MHFA) program which aims to increase public mental health literacy. We conducted this program at a company workplace among 91 employees, and ultimately 83 participants completed the self-rated questionnaires. Changes in confidence and practical skills in early intervention of depression and suicide-prevention, and stigma toward mental health problems were evaluated using self-rated questionnaires at 3 time-points; pre-program, immediately post-program, and 1 month after the program.

**Results:**

Confidence and practical skills were significantly improved even 1 month after the program, and stigma reduced just after the program.

**Conclusions:**

Our pilot study suggests that the program has a positive impact on encouraging employees to support their co-workers with mental health problems, and is applicable for busy workers due to its short duration. A single-arm design, evaluation using self-rated questionnaire and short-term follow up period are the main limitations of the present study. Hence, future research is required to validate the effects of this program with control groups, and also to assess long-term effectiveness and objective changes such as absenteeism and sick leave.

**Trial registration:**

UMIN Clinical Trials Registry (UMIN-CTR) R000023258

## Introduction

Depression and suicide are serious mental health problems that greatly impact the workplace. According to the World Mental Health Japan (WMH-J) survey, the most frequent mental health problem among Japanese employees is major depressive disorder, and its 12-month prevalence is estimated to be 2.6% [1]. In the workplace, depression leads to absenteeism, with an estimated associated cost to the Japanese economy of approximately $6 billion annually [2]. Although cases of suicide among employed individuals has decreased from 2010, the number of cases remains over 6000 per year [3]. On the other hand, a survey on the behavior of people suffering from mental health problems indicates that only 19% of the serious or moderate cases of major depressive disorder received medical treatment within the last 12 months [4], and hence, support by mental health professionals is prone to delay. It is clear that effective intervention targeting the individual with mental health problems in the workplace is warranted.

Mental Health First Aid (MHFA) is a training program developed in Australia in 2000. The purpose of the program is to increase public mental health literacy, including recognition and knowledge of mental health problems, help-seeking behavior, and providing help to someone with a mental health problem [5]. The standard MHFA program is a 12-h course applicable to diverse conditions including depression, anxiety disorders, psychosis, and substance use disorder [6]. The MHFA program is effective in improving knowledge, reducing stigmatizing attitudes, and increasing supportive behaviors [7]. This effectiveness has been examined in workplace settings using randomized controlled trials in several countries [8–10]. Furthermore, the MHFA guideline for office workers has recently been released [11], suggesting that workers are more comfortable to disclose a mental health problem if they and their co-workers have confidence and adequate skills in providing MHFA.

Although the standard MHFA program takes 12-h, shortened versions of program have been warranted to make such training more accessible to people for whom time may be a consideration. In Japan, a 2-h program was developed targeting medical staff and university administrative staff by focusing on depression and the risk of suicide and showed effectiveness in improving confidence and skills in managing persons with mental health problems, and attitudes toward psychiatric disorders [12–14]. To our knowledge, such brief programs based on the MHFA have never been developed for workplace settings. We have herein developed a novel educational training program for workplace settings based on our previous MHFA studies. The purpose of the present study is to evaluate the effectiveness of this program as a single-arm open trial in a workplace setting as a pilot study.

## Methods

This study was approved by the ethics committee of Kyushu University, and was registered at the UMIN Clinical Trials Registry (UMIN-CTR) (Registration number: R000023258).

### Participants

**Fig 1:**
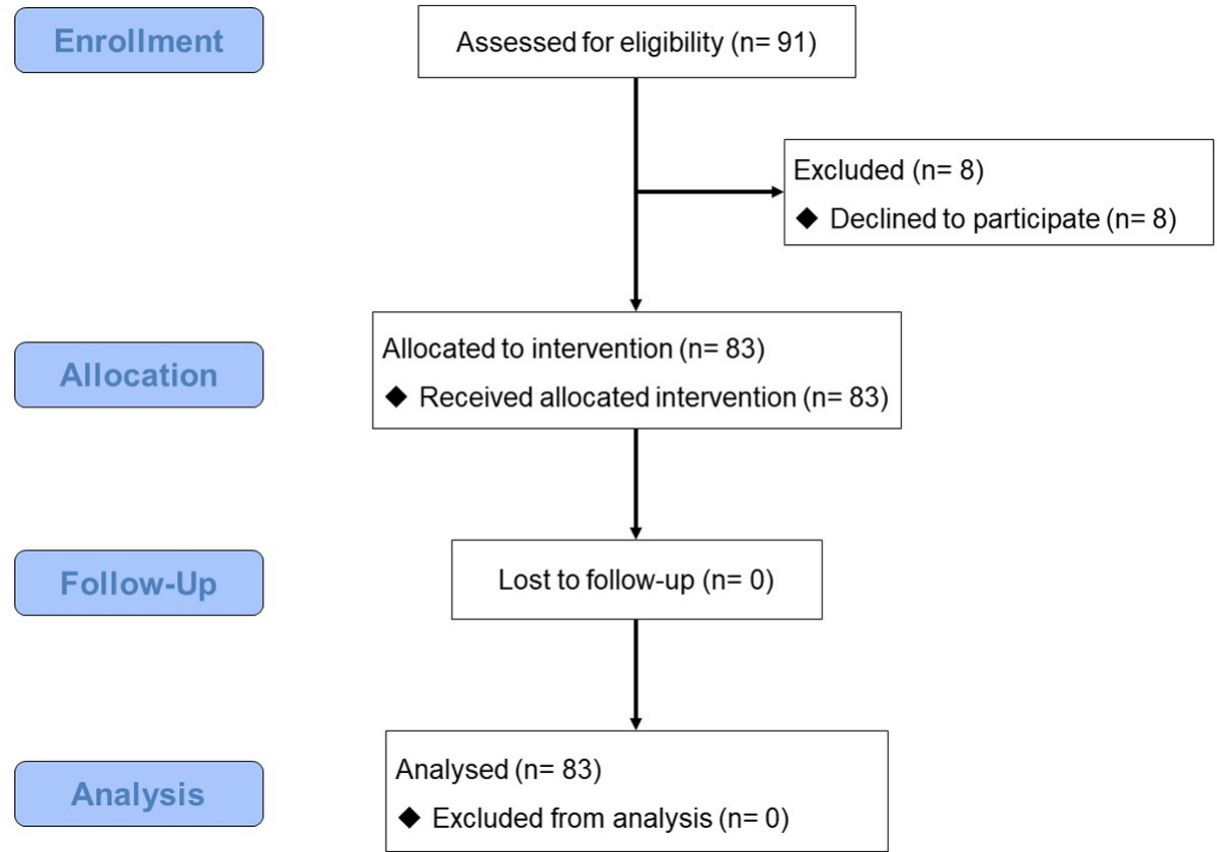
Participant flow chart.

91 employees working in a manufacturing company in western Japan attended a 2-h mandatory mental health literacy course during their work day. This course was held twice and employees attended either of the courses. The first course was held in June, and the second was held in July of 2016, the number of employees attending the course was 40 and 51, respectively. Employees were informed of the aims and methods of the present study and that their participation as whether or not to answer the self-rated questionnaires was completely voluntary. Participants who agreed to join this study were then registered as participants with written informed consent. 39 out of 40 employees participated in the first course, and 44 out of 51 in the second course. Finally, 83 employees participated in this study. The participant flow chart is shown in Fig 1. Both courses were conducted exactly the same way by an experienced psychiatrist who is a trainer of the MHFA-Japan (T.A.K.).

### Program development

This training program was established based on the standard MHFA program which is a 12-h course consisted of lectures and workshops focusing on the following 5-step MHFA principles: 1) Approach the person, assess and assist with any crisis; 2) Listen non-judgmentally; 3) Give support and information; 4) Encourage the person to get appropriate professional help; and 5) Encourage other support [6]. Based on this 12-h MHFA program, we previously developed a shorter 2-h educational program for clinical settings especially focusing on dealing with people with depression and suicidal risk [12, 14]. In the present study, the training program for workplace settings was developed through modifications of the 2-h program for clinical settings. The main points of modifications are shown as follows:

1. The previous lecture and role-play sessions for clinical settings was modified including audiovisual material (DVD) and role-play scenario enabling workers to learn how to approach and respond to co-workers with depression and suicidal thoughts.
2. At the beginning of the program, a 15-min role-play session where participants were required to approach a person (co-worker) with depression without any knowledge or skills of MHFA was introduced in order for participants to recognize the difficulty in dealing with such a person. After the introductory session, topics relevant to supporting such people were briefly discussed (e.g. appropriate time and place to approach while maintaining the person’s confidentiality and privacy, information regarding professional support such as occupational health physicians, nurses and so on).
3. In the training program, we emphasized the difficulty in approaching a person with depression in the workplace which was partly due to Japanese workplace culture and/or stigma. Recent topics on mental health in the workplace were included. In particular, the Stress Check Program and modern-type depression in Japan were introduced. The Stress Check Program is an occupational policy program started in 2015, aiming at screening for workers with high psychological stress by questionnaires assessing mental health problems. The results of the questionnaire are informed to workers individually, then a physician’s interview is arranged. In addition, this program requires employers to make efforts to improve the workplace environment [15]. In addition, sociocultural issues of modern-type depression, a novel form of depression-related syndromes increasing among Japanese youth especially in workplace settings, was introduced [16–21]. The content of the 2-h training program for workplace settings is shown in Table 1.

**Table 1:** 2-h educational program for early intervention in depression among office workers.

I. Introduction (15 min)
At the beginning of the session participants watched a 3-min DVD, in which a co-worker responds to a person with depression in an inadequate way. Without using any scenario, a pair of participants played the role of a co-worker and a person with depression alternately to experience the difficulty in approaching persons with depression.
II. Lecture (50 min)
The following parts were highlighted to improve participants’ knowledge about depression and suicide.
1) Risk factors and signs of depression and suicide.
2) Recent topics of Stress Check Program and Modern Type Depression in Japan.
3) Importance of identifying and managing co-worker’s risk with paying attention to stigma and workplace culture.
4) Instructors who took the MHFA training course in Melbourne taught early intervention skills based on the five-step principles of MHFA, which were modified to workplace settings.
Five-step principles of the MHFA (3rd version)
Step 1) Approach the person, assess and assist with any crisis
Step 2) Listen non-judgmentally
Step 3) Give support and information
Step 4) Encourage the person to get appropriate professional help
Step 5) Encourage other support
III. Role-play (Step 2 of the MHFA principles): Listening skills (15 min)
A pair of participants played a listener and a speaker role alternately, in order to acquire skills needed for listening non-judgmentally.
IV. MHFA Role-play (Step 1, 3, 4 and 5 of the MHFA principles) (30 min)
Participants watched a 5-min DVD, in which a co-worker responds to a person with depression ideally using the five-step principles of the MHFA. Then, a pair of participants played the role of a co-worker and a person with depression using a workplace scenario, alternately.
V. Q&A time (10 min)

### Measurements

To evaluate the effectiveness of the training program, a questionnaire assessing respondents’ confidence and skills in dealing with people with depression and suicidal risk based on the MHFA, and stigma toward mental health problems was conducted. The same questionnaire was conducted anonymously at all 3 time points (pre-program, immediately post-program, 1 month post-program). In addition, at pre-program, participant demographic data were collected (age, gender and occupation).

#### 1. Confidence

The confidence level in management of people with depression and/or suicidal risk was evaluated by 6 questions based on the MHFA strategy; 1) approaching a person with symptoms of depression, 2) listening non-judgmentally, 3) telling the possibility of clinical depression, 4) giving support and information, 5) asking “suicidal thoughts”, 6) encouraging the person to obtain appropriate professional help. Participants were asked to answer these questions on a 5-point Likert scale, ranging from 1 (not confident at all) to 5 (very confident).

#### 2. Skill

To evaluate practical skills in early intervention of depression and suicide-prevention in the workplace, original questionnaires using a case vignette was developed as shown below:

Ms. M is a 30-year-old office worker. Formally, she was a bright and sociable person, over the last few weeks she seems to be feeling sadness and unhappiness in a way that she has never experienced before. She always looks tired and drowsy in the workplace. She appears not to sleep well. She has no appetite and is clearly losing weight. She can’t concentrate on her tasks at work, and she tends to make easy mistakes. Her boss considers Ms. M’s current condition as concerning.

You (respondent), Ms. M’s co-worker, are concerned about Ms. M’s current condition too.

Imagine you are a co-worker of Ms. M, would you deal with Ms. M in the following way or not?

Participants (respondents) are asked to imagine themselves as co-worker of Ms. M, and to answer 10 questions rating the possibility of performing behaviors; each question was developed to evaluate skills for early intervention of depression and suicide-prevention based on the MHFA strategy. The possibility of each behavior was rated using 5-point Likert scale, indicating 0 (absolutely no), 1 (probably not), 2 (don’t know), 3 (probably yes), and 4 (absolutely yes).

#### 3. Stigma

The Japanese version of Link’s Devaluation-Discrimination Scale was conducted to measure respondents’ stigma toward mental health problems, by asking respondents to appraise their feelings toward people who have experienced psychiatric treatment. This scale is consisted of 12 items with a 4-point Likert scale, ranging from 1 (strongly disagree) to 4 (strongly agree), the higher the total score, the higher the stigma the respondent feels. Reliability and validity of the Japanese version scale were confirmed [22]. Average score of general Japanese people is reported to be 31.95 (SD = 5.74) [22].

### Statistics

All analysis was performed using IBM SPSS 23 Advanced Statistics for Mac OS. Results of confidence level, skills, and stigma were compared at pre-program, post-program and 1 month post-program. Comparisons were performed using total participant data (namely, 83 participants). Shapiro-Wilk test was used to evaluate the normality of data, and revealed non-normal distribution except for total score of Link’s Devaluation-Discrimination Scale. A one-way repeated measures analysis of variance (ANOVA) is known to be reasonably accurate even with non-normal distribution data [23], thus we used ANOVA to identify any significant difference over time. Post hoc comparison tests were performed in order to compare the data of pre-program, post-program and 1 month post-program, when an ANOVA model indicated a significant difference. The value of p < 0.017 using the Bonferroni correction for multiple comparisons was considered indicative of statistical significance.

## Results

### Demographic data of participants

We conducted our newly developed program at a company workplace twice among 91 employees, and 83 participants were agreed to participate in this study. All the participants completed the self-rated questionnaires at 3 time points (pre-program, post-program and 1 month after the program). The basic characteristics of participants were shown in Table 2. Among the participants, 77.1% were males. As for type of occupation, over half of participants were engaged in research and development or technical divisions.

**Table 2:** Basic characteristics of participants.

Total number of participants	n = 83
Male, n	64
Female, n	19
Mean of age (S.D.)	29.00 (4.21)

### Confidence

Self-confidence in dealing with people with depression and/or the risk of suicide based on the MHFA was ascertained in 6 areas. Changes in participants’ confidence were shown in Table 3. Confidence in all 6 areas was significantly improved even 1 month after the program.

**Table 3:** Confidence level in management of people with depression and suicidal risk.

Number of participants		Pre	Post	1-month later	p-value (Repeated ANOVA)	Significance (ANOVA)
**1. Are you confident to talk to a person with depressive symptoms to initiate the support? [Step 1: Approach the person, assess and assist with any crisis]**						
**n = 79**	**Mean**	**0.99**	**1.94**	**1.72**	**< 0.001**	**Pre < Post, Pre < 1-month later, Post > 1-month later**
	SD	0.71	0.74	0.62		
**2. Are you confident to listen to a person with depressive symptoms at his/her own pace? [Step 2: Listen non-judgmentally]**						
**n = 79**	**Mean**	**1.42**	**2.20**	**2.03**	**< 0.001**	**Pre < Post, Pre < 1-month later**
	SD	0.71	0.69	0.70		
**3. Are you confident to directly convey the possibility of clinical depression to a person with depressive symptoms? [Step 3: Give support and information]**						
**n = 79**	**Mean**	**1.10**	**1.79**	**1.73**	**< 0.001**	**Pre < Post, Pre < 1-month later**
	SD	1.01	0.83	0.87		
**4. Are you confident to give information regarding depression to a person with depressive symptoms? [Step 3: Give support and information]**						
**n = 79**	**Mean**	**1.19**	**1.94**	**1.82**	**< 0.001**	**Pre < Post, Pre < 1-month later**
	SD	0.95	0.77	0.76		
**5. Are you confident to directly ask a person with depressive symptoms whether they have suicidal thoughts? [Step 3: Give support and information]**						
**n = 79**	**Mean**	**0.72**	**1.42**	**1.28**	**< 0.001**	**Pre < Post, Pre < 1-month later**
	SD	0.89	0.83	0.85		
**6. Are you confident to recommend a person with depressive symptoms to the support by professionals? [Step 4: Encourage the person to get appropriate professional help]**						
**n = 79**	**Mean**	**1.57**	**2.28**	**2.14**	**< 0.001**	**Pre < Post, Pre < 1-month later**
	SD	0.96	0.80	0.78		

ANOVA = analysis of variance

### Skill

Practical skills based on the MHFA were evaluated by 10 questions using an original case vignette (shown in Table 4). 6 out of 10 scores were significantly improved even 1 month after the program, including “asking the person whether he/she is in any trouble,” “telling probability of depression,” “informing the person of depression,” “asking suicidal thoughts,” and “recommending the person to visit a psychiatric or psychosomatic department.” These skills are associated with MHFA strategy of “Approach the person, assess and assist with any crisis,” “Give support and information,” and “Encourage the person to get appropriate professional help.”

**Table 4:** Skills of supporting people with depression and suicidal risk (responses to a workplace case vignette).

Number of participants		Pre	Post	1-month later	p-value (Repeated ANOVA)	Significance (ANOVA)
**1. Do you leave Ms. M alone for a while without saying anything? [Step 1: Approach the person, assess and assist with any crisis (This is not an appropriate behavior)]**						
**n = 81**	**Mean**	**1.47**	**1.12**	**1.47**	**0.007**	**Pre > Post, Post < 1-month later**
	SD	0.99	1.02	1.01		
**2. Do you directly ask Ms. M “Are you in any trouble?” at an early stage? [Step 1: Approach the person, assess and assist with any crisis]**						
**n = 80**	**Mean**	**2.54**	**2.94**	**2.81**	**0.002**	**Pre < Post, Pre < 1-month later**
	SD	1.04	0.74	0.70		
**3. Do you consult with someone (supervisor, colleague, or occupational health nurse, etc.) about supporting Ms. M? [Step 5: Encourage other support]**						
**n = 81**	**Mean**	**2.52**	**2.91**	**2.77**	**0.001**	**Pre < Post**
	SD	1.04	0.71	0.83		
**4. Do you suggest Ms. M for the way to release her stress? [Step 5: Encourage other support]**						
**n = 81**	**Mean**	**2.02**	**2.23**	**2.31**	**0.143**	**――**
	SD	1.41	1.03	1.00		
**5. Do you think it a good way to directly convey the possibility of clinical depression to Ms. M when her depressed mood proved to be serious from conversation? [Step 3: Give support and information]**						
**n = 81**	**Mean**	**1.63**	**2.80**	**2.64**	**< 0.001**	**Pre < Post, Pre < 1-month later**
	SD	1.11	0.81	0.75		
**6. Do you explain to Ms. M about the depression? [Step 3: Give support and information]**						
**n = 80**	**Mean**	**1.28**	**2.60**	**2.38**	**< 0.001**	**Pre < Post, Pre < 1-month later**
	SD	1.08	0.91	0.89		
**7. When you think Ms. M probably got depression, do you ask her if she has ‘suicidal thoughts’? [Step 3: Give support and information]**						
**n = 81**	**Mean**	**0.95**	**2.01**	**1.79**	**< 0.001**	**Pre < Post, Pre < 1-month later**
	SD	1.01	0.97	1.02		
**8. Do you think it a good way to directly ask Ms. M about her ‘suicidal thoughts’? [Step 3: Give support and information]**						
**n = 81**	**Mean**	**1.22**	**2.36**	**2.23**	**< 0.001**	**Pre < Post, Pre < 1-month later**
	SD	0.73	0.73	0.73		
**9. Do you directly recommend that Ms. M visit a psychiatric or psychosomatic department? [Step 4: Encourage the person to get appropriate professional help]**						
**n = 80**	**Mean**	**2.00**	**2.84**	**2.60**	**< 0.001**	**Pre < Post, Pre < 1-month later, Post > 1-month later**
	SD	1.08	0.80	0.79		
**10. Do you say to Ms. M, “Even you feel hard now, you will get well”? [Step 3: Give support and information]**						
**n = 81**	**Mean**	**1.64**	**1.95**	**2.07**	**0.005**	**Pre < 1-month later**
	SD	1.22	1.24	1.09		

### Stigma

Stigma toward psychiatric disorders was assessed by the Japanese version of Link’s Devaluation-Discrimination Scale. The total score was shown in Table 5. The total score significantly decreased just after the program, however, a significant difference was not observed 1 month after the program.

**Table 5:** Stigma toward psychiatric disorders (total scores of Japanese version of Link’s Devaluation-Discrimination Scale).

Number of participants		Pre	Post	1-month later	p-value (Repeated ANOVA)	Significance (ANOVA)
**n = 72**	**Mean**	**28.29**	**26.11**	**27.26**	**0.003**	**Pre > Post**
	SD	4.90	5.36	5.78		

## Discussion

In the present study, we newly developed a brief educational training program for workplace settings based on the MHFA, and evaluated the effectiveness of this program. We found that the brief program was effective in improving confidence in all areas required under the MHFA 5-step principles. Regarding practical skills in management of people with depression or suicidal thoughts, we have revealed that the program has a positive impact on some skills. These skills include dealing with situations utilizing active and direct communications such as “directly ask the person about suicidal thoughts” or “directly tell the probability of depression.” In general, even if an employee experiences mental health problems, his/her co-workers tend not to recognize the occurrence of the problem or feel unwilling to offer help with the employee’s mental health problems due to inadequate knowledge [24]. Therefore, we believe that the present program encourages individuals to support colleagues facing mental health issues.

Previous MHFA-based training courses for workplaces took 9 or 12 hours [8–10, 25]. The standard MHFA program deals with a variety of mental health problems including depression, anxiety, psychosis, and substance use disorder [6]. We have limited our program to 2-h by focusing on depression and suicidal thoughts, which are known to be highlighted mental health issues in Japanese workplaces [1]. Our pilot study suggests that this program is applicable even for busy workers due to its short duration.

Besides the lack of knowledge of mental health problems, stigma is one of the most important barriers affecting the delay in seeking professional help in the workplace. For example, individuals suffering from mental health problems feel “embarrassed” when people around him/her come to know about their mental health problems specifically in young employees [26]. Furthermore, employees who perceive risk for damaging their careers or losing future opportunities apt to hesitate in seeking professional help [27]. Importantly, a survey reported that 62.5% of people with major depression had anticipated and/or experienced discrimination in their workplaces, suggesting the necessity of interventions to decrease stigma in the workplace [28]. Employees tend to reveal their mental health problems when the workplace climate or culture is supportive and free from risk of social exclusion [27]. The present program showed effectiveness in reducing stigma just after the program, however, long-term effectiveness was not observed. Respondents with a total score more than 30 are reported to have higher stigma toward people with mental health problems [29]. In the present study, the average score of the scale was 28.29 at pre-test. The score indicates that the level of stigma among participants is not high, therefore, the present results regarding the effectiveness in reducing stigma should be carefully considered. To reveal the long-term effect on reducing stigma, further research is needed including groups with high stigma toward people with mental health problems.

Previous systematic reviews on intervention programs targeting workplace settings pointed out the difficulty in drawing general conclusions of effectiveness of the intervention owing to the broad diversity of research [25, 30–33]. In Japan, the Ministry of Health, Labor, and Welfare (MHLW) published “Guidelines for the Maintenance and Promotion of the Mental Health of Workers” to tackle mental health problems in the workplace [34]. The guideline emphasizes the importance of four areas of “care”; “self-care by the employees themselves,” “line-care by line managers,” “care by occupational health care staff” and “care by special facilities outside of organization.” Our program may be useful in “self-care” via improving awareness of mental health problems and seeking professional help by employees themselves. In addition, the present program is effective for obtaining knowledge and skills in management of depressive symptoms and suicidal risk, and possibly effective in reducing stigma toward psychiatric disorders through role-playing which encourages non-judgmental listening. Previous research showed effectiveness of supervisory education programs utilizing the combination of lecture and active listening, suggesting that providing supervisors with useful skills has a positive effect on subordinates’ psychological well-being [35, 36]. The present program includes the contents relevant to active listening, thus, the program may be a prospective tool for “line-care” by managers. Furthermore, we suggest that the present program encourages employees to support their co-workers with mental health problems, resulting in the creation of supportive atmosphere in the workplace.

### Future perspectives

There are some limitations in our study. First, we utilized self-rated questionnaires, which might not reflect participants’ actual attitudes and behaviors. In evaluations, assessment of objective outcomes such as reduction in absenteeism or sick leave, and increase in utilization of mental health services among employees is needed. Regarding the skill assessments using responses of the case vignette, we have evaluated step 1, 3, 4 and 5 of the MHFA five-step skills (Table 4). We have not included step 2 (Listen non-judgmentally) in the skill assessment questions assuming that listening skill was impractical to assess using a self-rated questionnaire. We introduced a 15-min role-play session where participants play a listener and a speaker role alternately, in order to acquire skills needed for listening non-judgmentally. We believe that such a listening role-play session is useful for participant to obtain practical skills, and it is worthwhile assessing listening skills using questionnaires in a future study. In addition, our study is limited to short-term follow up; assessment of long-term effectiveness is required. In the present pilot study, we conducted parametric statistical analysis in accordance with our previous study [14]. However, the present data was non-normal distribution data, thus ideally, non-parametric statistical analysis is the most desirable method. Therefore, some significant outcomes in the present study based on parametric statistical analysis should carefully be considered in drawing general conclusion regarding the effectiveness of the present program. To combat this limitation, we should include a far greater number of participants in our future trial so as to obtain a normal distribution data set. Finally, the study design was a single-arm trial with no control groups. To validate the effectiveness of our newly developed program, a randomized controlled trial with a longer follow up period is required as future research.

## Supporting information

S1 File TREND checklist. (PDF)

S2 File Study protocol (translation version) revised. (PDF)

S3 File Study protocol (Japanese version) revised. (PDF)
