# Supplementary material for: Development of MHFA-based 2-h educational program for early intervention in depression among office workers: A single-arm pilot trial
Source: PLoS One. 2018 Dec 7;13(12):e0208114. doi: 10.1371/journal.pone.0208114 (PMC6285460; doi:10.1371/journal.pone.0208114)
Supplement: S2 File — (PDF) [file pone.0208114.s002.pdf]

Translated at 2018/05/01

# Study protocol

## 0. Title of the study

Development of Educational Program for Early Intervention of Mental Illnesses based on the Mental Health First Aid (MHFA) among medical practitioners: Multicenter pilot study

## 1. Purpose of the study

To evaluate the effectiveness of a brief structured training program based on Mental Health First Aid to increase knowledge and skills for medical staff, welfare service workers, and non-medical staff such as office workers in order to deal with persons with depression and suicidal thoughts.

## 2. Background and objectives

People with mental health problems such as depression tend not to visit psychiatry or psychosomatic clinics/hospitals as their first choice. Majority of them initially complain about somatic symptoms and tend to visit internal medicine clines and so on for somatic symptoms. Thus, adequate psychiatric interventions tend to be delayed. It is important for non-psychiatric professionals such as physicians, nurses and/or office workers to obtain knowledge of mental health problems and skills of early intervention.

Mental Health First Aid (MHFA) is a 12-h educational/training program developed in Australia in 2000. The purpose of the program is to increase public mental health literacy, including recognition and knowledge of mental health problems, help-seeking behavior, and providing help to someone with a mental health problem [1-2].

We have engaged in spreading the MHFA program in Japan. In addition, we have recently developed a 2-h short educational program targeting medical staff and university administrative staff by limiting the topics only about depression and suicide intervention. We have shown the effectiveness of the program in improving confidence and skills in managing persons with mental health problems, and attitudes toward psychiatric disorders [3-5]. We have continued to improve the 2-h program according to the demands of the present mental health issues.

In the present study, we will evaluate the effectiveness of the novel 2-h brief educational programs based on the MHFA as a single-arm multi-centered pilot study. These programs include several versions targeting medical staff (doctors, nurses, etc.), welfare staff, medical/nurse students, or office workers. We will assess the change of score of the questionnaire asking knowledge, skills and confidence to deal

with persons with depression and suicidal thoughts at just before, just after and 1 month later the intervention.

[References]
1) Kitchener BA, Jorm AF: Mental health first aid training: review of evaluation studies. Aust N Z J Psychiatry. 40: 6-8. (2006).
2) Jorm AF, Blewitt KA, Griffiths KM, Kitchener BA, Parslow RA: Mental health first aid responses of the public: results from an Australian national survey. BMC Psychiatry. 5: 9, (2005).
3) Kato TA, Suzuki Y, Sato R, Fujisawa D, Uehara K, Hashimoto N, Sawayama Y, Hayashi J, Kanba S, Otsuka K: Development of two-hour suicide intervention program among medical residents: First pilot trial. Psychiatry Clin Neurosci 64(5): 531-540. (2010).
4) Otsuka K, Suzuki Y, Fujisawa D, Kato TA, Sato R, Aoyama-Uehara K, Hashimoto N, Suzuki S, Kurosawa M: The activities of Mental Health First Aid-Japan Team. Seishin Shinkeigaku Zasshi. 115(7):792-6. (2013). (in Japanese)
5) Suzuki Y, Kato TA, Sato R, Fujisawa D, Aoyama-Uehara K, Hashimoto N, Yonemoto N, Fukasawa M, Otsuka K: Effectiveness of brief suicide management training program for medical residents in Japan: A cluster randomized controlled trial. Epidemiol Psychiatr Sci. 23(2): 167-76. (2014).

## 3. Participants

Target sample size: 350 (Kyushu University Hospital: 60, Iwate Medical University Hospital: 20, Keio University Hospital: 20, Hokkaido University Hospital: 20, Yokohama City University Hospital: 20, Kitakyushu Mental Health Center: 40, Iizuka Hospital: 60, Kyoto University Hospital: 60, Non-medical facility (such as a private company: 50)

Eligibility

Inclusion criteria

1) Medical staff (Medical doctors, medical residents, nurses, pharmacists, and their students), welfare service workers, non-medical staff working at non-medical faculties such as student-counseling centers and general companies. All participants need to have written informed consents on this study
2) Not under the age of 18 years old

Exclusion criteria

1) Insufficient literacy of Japanese language

Cancellation criteria

1) Under 80% of participation for total time of the program
2) Not participating in any role-play sessions

## 4. Methods

Participants who agree to join this study are then registered as participants with written informed consent. We implement 4 types of 2-h brief educational program, namely, A) Doctor version (for medical doctors and medical residents) B) Nurse version (for nurses and nurse students) C) Other medical version (for other medical staff and welfare workers) D) Non-medical version (for other non-medical professionals such as office workers). Doctor version is mainly implemented at Kyushu University, Iwate Medical University, Keio University, Yokohama City University, and Iizuka Hospital. Nurse version is mainly implemented at Kyoto University. Other medical version is mainly implemented at Hokkaido University and Kitakyushu Mental Health Center. Non-medical version is implemented at companies in Fukuoka prefecture. Study outline is shown in Figure 1.

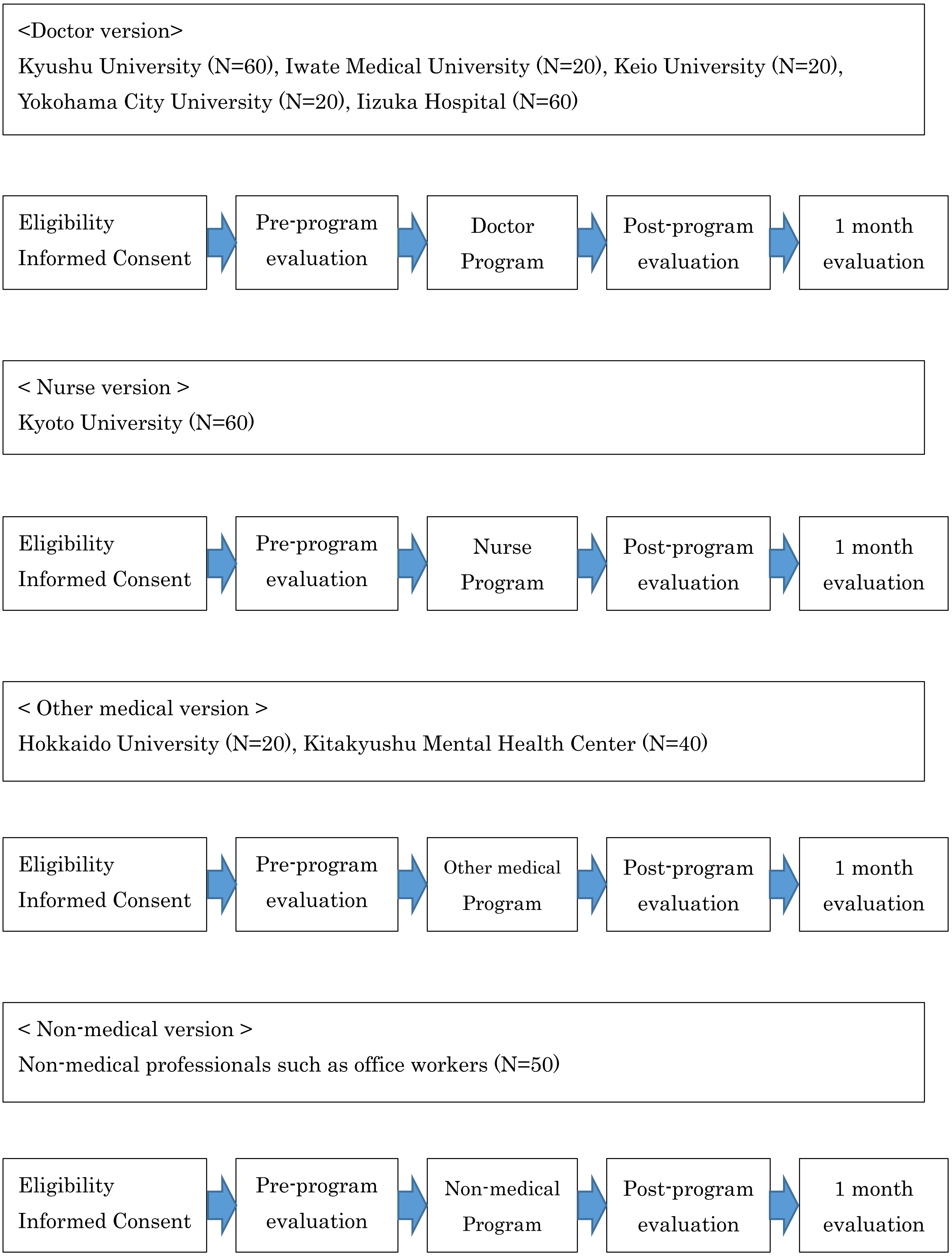


Figure 1: Outline of the study

＜Content of the program＞

This program has been established based on the standard MHFA program which is a 12-h course consisted of lectures and workshops focusing on the following 5-step MHFA principles: 1) Approach the person, assess and assist with any crisis; 2) Listen non-judgmentally; 3) Give support and information; 4) Encourage the person to get appropriate professional help; and 5) Encourage other support. Based on this 12-h MHFA program, we have developed a shorter 2-h educational program especially focusing on dealing with people with depression and suicidal risk.

[Lecture]

Lecture includes following contents;

- What is MHFA?
- Knowledge and skills to deal with persons with depression and/or suicidal risk
- 5-step principles of the MHFA

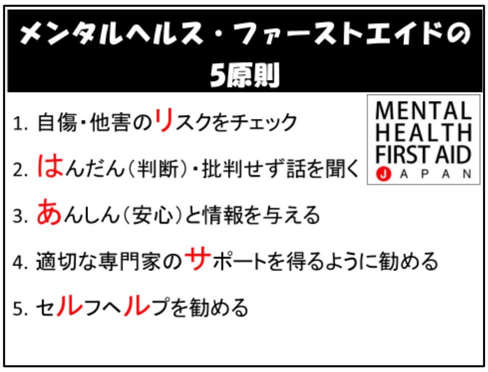


Fig 2.　5-step of MHFA

[DVD material]

At the beginning of the lecture session, participants watch a DVD, in which a person responds to a person with depression (a clinical patient or a co-worker) in inadequate ways. After taking the lecture, participants watch a DVD, in which a person responds to a person with depression ideally using the five-step principles of the MHFA.

[MHFA Role-play]

MHFA Role-play is consisted of 2 types of role-play; (1) Listening and Scenario role-play. In the Listening, A pair of participants played a listener and a speaker role alternately, in order to acquire skills needed for listening non-judgmentally. (2) After watching DVD (an ideal version), a pair of participants need to play the role of a supporting person and a person with depression using a scenario.

Each program includes specific contents due to the differences of occupational backgrounds.

【Doctor version】

・Psychiatric knowledge to recognize the mental health problems

・Detailed skills for referring the patient to mental health professionals

【Nurse version】

・How to observe mental health problems

・How to communicate with patients in safer way

【Other medical version】
・How to recognize mental health problems in their specific occupations
・Skills in giving information in their specific occupations

【Non-medical version】
・How to communicate with persons with mental health problems
・To reduce the stigma toward mental health problems

＜Implementation of the program and evaluation＞

To evaluate the effectiveness of the educational/training program, a self-rated questionnaire assessing respondents' confidence and skills in dealing with people with depression and suicidal risk based on the MHFA, and stigma toward mental health problems was conducted. The same questionnaire was conducted anonymously at all 3 time points (pre-program, immediately post-program, 1 month post-program). In addition, at pre-program, participant demographic data were collected (age, gender and occupational backgrounds).

① Pre-program evaluation:

Just before the program, participants are required to answer the questionnaire. The questionnaire is consisted of following 6 items.

1) Demographic data (age, gender, occupation, experience of supporting a person with mental health problem)
2) Practical skills: original questionnaires with 10 items using a case vignette
3) Confidence: the confidence level in management of people with depression and/or suicidal risk was evaluated by 6 questions based on the MHFA strategy
4) Behavior: Actual supporting of people with mental health problems within last 1 month
5) Attitude toward the case of committing suicide: The Understanding Suicidal Patients (USP) scale
6) Stigma toward mental health problems: the Japanese version of Link's Devaluation-Discrimination Scale

#. USP scale (item 5) is targeting medical professionals, therefore, participants who take non-medical version program are required to answer the Japanese version of Link's Devaluation-Discrimination Scale (item 6) in spite of USP scale.

②Conducting the program and immediately post-program evaluation:

Participants took one of the following programs in accordance with their occupational type:

A) Doctor version
B) Nurse version
C) Other medical version
D) Non-medical version

Immediately after the program, participants are asked to answer the questionnaire including the above 6 items. In addition, participants are asked to give any demands to improve the program and to answer how they were satisfied with the program.

③1 month post-program evaluation:

After 1 month of the program, participants are asked to answer the questionnaire (identical to pre-program) via mailing or on-line forms.

## 5. Schedule of the study

From approved date to 31st March, 2019

## 6. Benefit and Disadvantage

Benefit:

By attending this program, participants are expected to improve the skills to deal with persons with mental health programs and to increase mental health literacy.

Disadvantage:

We believe that this study is minimally invasive because the program is consisted of lecture and workshop taking jut only 2 hours. However, some participants might feel anxiety during facing unfamiliar contents of the program. If participants have strong anxiety or have any other problems, PI and study conductors in the present study (including medical doctors) will do their best to deal with such problems.

## 7. Outcomes

**Primary:**

- Changes in practical skills between pre-program and immediately post-program, and changes in practical skills between immediately post-program and 1 month post-program
- Changes in score of USP scale between pre-program and immediately post-program,

and changes in score of USP scale between immediately post-program and 1 month post-program

**Secondary:**

- Changes in confidence level between pre-program and immediately post-program, and changes in confidence level between immediately post-program and 1 month post-program
- Changes in score of the Japanese version of Link's Devaluation-Discrimination Scale between pre-program and immediately post-program, and changes in score of the Japanese version of Link's Devaluation-Discrimination Scale between immediately post-program and 1 month

## 8. Statistics

Target sample size: 350

Analysis:

As to the participants of each program, results of practical skills, score of USP scale, confidence level and score of the Japanese version of Link's Devaluation-Discrimination Scale were compared at pre-program, post-program and 1 month post-program.

## 9. Ethical issues

Participants will be informed of the aims and methods of the present study based on the "Declaration of Helsinki" and "Ethical Guidelines for Medical and Health Research Involving Human Subjects." Their participations as whether or not to answer the self-rated questionnaires will be completely voluntary. Participants who agree to join this study will be then registered as participants with written informed consent.

## 10. Management in adverse event

We believe that this study is minimally invasive because the program is consisted of lecture and workshop taking jut only 2 hours. However, some participants might feel anxiety during facing unfamiliar contents of the program. If participants have strong anxiety or have any other problems, PI and study conductors in the present study (including medical doctors) will do their best to deal with such problems.

## 11. Protection of personal information

To protect the personal information, anonymization will be conducted under the

responsibility of PI. When the data collected at collaborating institutions are sent to Kyushu University, anonymization of data is conducted under the responsibility of investigator of each institution. When a participant retracts the informed consent, his/her data is discarded.

## 12. Funding Source

This work is supported by a Grant-in-Aid for Scientific Research on (1) The Japan Agency for Medical Research and Development (AMED) (JP16dk0307028 & JP17dk0307073 to T.A.K.), and (2) KAKENHI - the Japan Society for the Promotion of Science (JP16H06403 to T.A.K. & JP25461781 to K.O.).

## 13. Competing Interests

In conducting the study, no competing interests exist.

## 14. Secondary use of the data

Data collected in the study is conserved for 5 years at Department of Neuropsychiatry, Graduate School of Medical Sciences, Kyushu University. If the participant consents to the secondary use of his/her data in writing, his/her data is conserved over 5 years for future studies. Prior to secondary use, any future studies should be approved by IRB.

## 15. Study organization

Principle investigator:

Shigenobu Kanba (Department of Neuropsychiatry, Graduate School of Medical Sciences, Kyushu University)

Co-principle Investigator/Study conductor:

Takahiro A. Kato (Department of Neuropsychiatry, Graduate School of Medical Sciences, Kyushu University)

Co-Investigators:

Koichi Akashi (Kyushu University Hospital Clinical Education Center/Department of Medicine and Biosystemic Science, Graduate School of Medical Sciences, Kyushu University)

Hiroaki Kubo (Department of Neuropsychiatry, Graduate School of Medical Sciences, Kyushu University)

Ryoko Katsuki (Department of Neuropsychiatry, Graduate School of Medical Sciences, Kyushu University)

Kohei Hayakawa (Department of Neuropsychiatry, Graduate School of Medical Sciences, Kyushu University)

Mina Kasai-Sato (Department of Neuropsychiatry, Graduate School of Medical Sciences, Kyushu University)

Norihiro Shimokawa (Department of Neuropsychiatry, Graduate School of Medical Sciences, Kyushu University)

Yoshie Kaneko (Department of Neuropsychiatry, Graduate School of Medical Sciences, Kyushu University)

Nobuki Kuwano (Department of Neuropsychiatry, Graduate School of Medical Sciences, Kyushu University)

Collaborators:

Kotaro Otsuka (Department of Neuropsychiatry, School of Medicine, Iwate Medical University)

Yuriko Suzuki (Department of Adult Mental Health, National Institute of Mental Health, National Center of Neurology and Psychiatry)

Daisuke Fujisawa (Department of Neuropsychiatry, Keio University School of Medicine)

Naoki Hashimoto (Department of Psychiatry, Hokkaido University Graduate School of Medicine)

Kumi Aoyama (Yokohama City University Hospital, Child Psychiatry)

Toshiko Mitsui (Kitakyushu Mental Health Center)

Hiroyuki Oda (Iizuka Hospital, General department)

Genichi Sugihara (Department of Psychiatry, Kyoto University Graduate School of Medicine)

Yukako Nakagami (Department of Psychiatry, Kyoto University Graduate School of Medicine)

Contact information (Study Office):

Kyushu University Hospital, Psychiatry

Contact officer: Takahiro A. Kato, MD, PhD

E-mail : takahiro@npsych.med.kyushu-u.ac.jp
