# Supplementary material for: Development of MHFA-based 2-h educational program for early intervention in depression among office workers: A single-arm pilot trial
Source: PLoS One. 2018 Dec 7;13(12):e0208114. doi: 10.1371/journal.pone.0208114 (PMC6285460; doi:10.1371/journal.pone.0208114)
Supplement: S3 File — (PDF) [file pone.0208114.s003.pdf]

研究計画書

0. 題目
「メンタルヘルス・ファーストエイドを応用した精神疾患患者早期介入のための医療従事者向け研修プログラム開発―多施設共同パイロット試験」

1. 研究目的
医療従事者（医師、研修医および医療専門職）および施設職員を対象に、メンタルヘルス・ファーストエイドに基づいて構造化された短期研修プログラムを実施し、プログラムの実施前後で参加者の精神医学的知識・スキルを測定し、研修プログラムの効果の検証を行う。本研究では、オーストラリアで開発され、米国では国家戦略となりつつあるメンタルヘルス・ファーストエイド（Mental Health First Aid: MHFA）を基盤として、我が国の医療従事者向けに開発した「精神疾患患者に対する早期介入のための教育研修プログラム」を、大学病院・総合病院を中心とした医療機関、保健センター、施設などにおいて実施し、研修プログラムの効果判定を行う多施設共同前向き研究を実施する。

2. 研究（試験）の背景と意義
うつ病など精神疾患をもつ患者が、最初から精神科・心療内科を受診することは稀である。多くは身体症状などを訴えて身体科を受診するため、適切な精神医学的対応は遅れがちで、慢性化や症状増悪、あるいは、自殺への対応の遅れが懸念される。しかるに、精神医療を専門としない医療従事者・施設職員などが精神疾患に関する知識や精神疾患患者への早期対応法を習得することは、精神疾患患者の早期治療に直結するため、厚生労働行政上急務である。

オーストラリアでは、精神疾患をもつ患者への初期対処法や精神保健知識を習得するために、精神疾患患者に対応する可能性の高い人々（消防、救急隊、聖職者など）や一般市民を対象に、うつ・自殺念慮など地域生活において直面する可能性のある精神状態像にどのように初期対応し、その後円滑に専門家の支援につなげるかを実践的に習得できる研修プログラムであるメンタルヘルス・ファーストエイド（Mental Health First Aid: MHFA ）が開発された。MHFA は数万人規模で普及し、その効果が量的研究、質的研究の両面で実証されている。

申請者らは、2007 年メルボルンにて MHFA の創始者である Kitchener B から直接プログラムを受講し、MHFA を日本語に翻訳した。その後 MHFA を基にした精神保健に関する短期研修プログラムを我が国で実施し、普及啓蒙活動に従事してきた。日本初の MHFA 講習会は、申請代表者の所属する九州大学病院にて 2008 年 4 月に初期臨床研修医オリエンテーションの中で 2 時間コースとして実施され、前向き研究にて臨床研修医の知識、スキル、自信が向上したことを明らかにした（Kato ら 2010）。申請者らは、内閣府ゲートキーパー養成研修プログラムの視覚教材やテキストなどの開発に学術的協力を行い、特に、内閣府自殺対策キャンペーンサイトや内閣府ホームページでは、視覚教材視聴や質問による学習内容

の確認などで構成される e-ラーニングの手法を取り入れることを提案し、採用された。こうした取り組みは東日本大震災の被災地支援向け教育内容にも採用されている。

申請者らは 2008 年以降、毎春の九州大学病院初期臨床研修医オリエンテーションに加えて、毎月精神科病棟にローテイトしてくる臨床研修医・医学生向けの MHFA 講習会を継続しており、本プログラムの有用性を実感するとともに、研修プログラムを本邦の医療現場の実情に合わせて開発、改訂を重ねてきた。

本研究では、申請者らがこれまで推進してきた MHFA 普及啓蒙活動の経験を踏まえて開発した「医療現場に特化した精神疾患患者への対応スキルが向上する研修プログラム」を、最大限の倫理的配慮の元で、全国の研究メンバーが所属する大学病院・総合病院・診療所・精神保健福祉センター・保健センターなどの医療従事者・施設職員を対象として実施し、その有効性をシングルアームで検証する。具体的には、医療従事者（医師、研修医および医療専門職）および施設職員などを対象に研修プログラムを実施し、評価項目として精神衛生への関心・知識・スキル等を尋ねる質問紙を、プログラム実施前後で実施することで、研修プログラムの有効性を検証する。加えて、プログラム実施 1 ヶ月後に同様の質問紙を実施し、研修プログラムの効果の持続性を検証する。

本研究における共同研究者は岩手医科大学医学部神経精神科学講座・教授・大塚耕太郎、国立精神・神経医療研究センター　精神保健研究所成人精神保健研究部・室長・鈴木友理子、慶應義塾大学医学部精神・神経科・専任講師・藤澤大介、北海道大学大学院医学研究科・客員研究員・橋本直樹、横浜市立大学附属病院児童精神科・助教・青山久美、北九州市精神保健福祉センター・所長・三井敏子、飯塚病院総合診療科・医師・小田浩之、京都大学医学部附属病院精神科神経科・院内講師・杉原　玄一、京都大学医学部附属病院精神科神経科・大学院生・中神由香子である。大塚にはプログラムにおける DVD 教材の監修、および、主に看護師向けの教育プログラム開発を依頼している。また、研修プログラムの実施を依頼している。鈴木には MHFA プログラムを実施できるインストラクターの養成、および、研究デザインを依頼している。藤澤には主に医師・研修医向けの教育プログラム開発を依頼している。また、研修プログラムの実施を依頼している。橋本、青山、三井、小田、杉原、中神には研修プログラムの実施を依頼している。

[参考文献]

1) Kato TA, Suzuki Y, Sato R, Fujisawa D, Uehara K, Hashimoto N, Sawayama Y, Hayashi J, Kanba S, Otsuka K: Development of two-hour suicide intervention program among medical residents: First pilot trial. Psychiatry Clin Neurosci 64(5): 531-540. (2010).
2) 大塚耕太郎, 鈴木友理子, 藤澤大介, 加藤隆弘, 佐藤玲子, 青山久美, 橋本直樹, 鈴木志麻子, 黒澤美枝: Mental Health First Aid-Japan チームの活動について. 精神神経学雑誌 115(7): 792-796. (2013).
3) Suzuki Y, Kato TA, Sato R, Fujisawa D, Aoyama-Uehara K, Hashimoto N, Yonemoto N, Fukasawa M, Otsuka K: Effectiveness of brief suicide management training program for medical residents in Japan: A cluster randomized controlled trial. Epidemiol Psychiatr Sci. 23(2): 167-76. (2014).

4) Kitchener BA, Jorm AF: Mental health first aid training: review of evaluation studies. Aust N Z J Psychiatry. 40: 6-8. (2006).
5) Jorm AF, Blewitt KA, Griffiths KM, Kitchener BA, Parslow RA: Mental health first aid responses of the public: results from an Australian national survey. BMC Psychiatry. 5: 9, (2005).

3. 参加者の選択あるいは対象

目標例数： 350 例
（九州大学病院 60 例、岩手医科大学附属病院 20 例、慶応義塾大学病院 20 例、北海道大学病院または北海道大学保健センター20 例、横浜市立大学附属病院 20 例、北九州市精神保健福祉センター40 例、飯塚病院 60 例、京都大学医学部附属病院 60 例、非医療機関（企業等）50 例）

適格基準
(1) 本研究への参加に関して同意が得られた医療従事者（医師、研修医および、看護師、薬剤師といった医療専門職、または医歯薬学・看護学といった医療関連領域を専攻する学生)、または保健・福祉関連の施設職員、ないし非医療機関職員（大学学生相談室、企業職員）
(2) 18 歳以上の男女

除外基準
(1) 日本語の読み書きが困難な者
(2) その他研究責任者または研究分担者が参加者として不適当と判断した者

中止基準とその手順
(1) 短期教育プログラムの総時間数の内、参加時間数が 80%未満の者
(2) 短期教育プログラムが定める演習のいずれかに参加しなかった者

4. 研究（試験）の方法

参加候補者に別添説明文書に基づき説明を行った上で、本研究への参加について同意を得る。参加者は、職種によって医師・研修医向けプログラム、看護師向けプログラム、その他医療職向けプログラム、非医療職向けプログラムに割り当てられる。医師・研修医向けプログラムは九州大学、岩手医科大学、慶應義塾大学、北海道大学、横浜市立大学、飯塚病院において実施する。看護師向けプログラムは京都大学において実施する。その他医療職向けプログラムは北海道大学保健センター、北九州市精神保健福祉センターにおいて実施する。非医療職向けプログラムは研修依頼のあった企業または非医療職向けプログラムが適切と判断された施設において実施する。研究の概略を次ページ図 1 に示す。

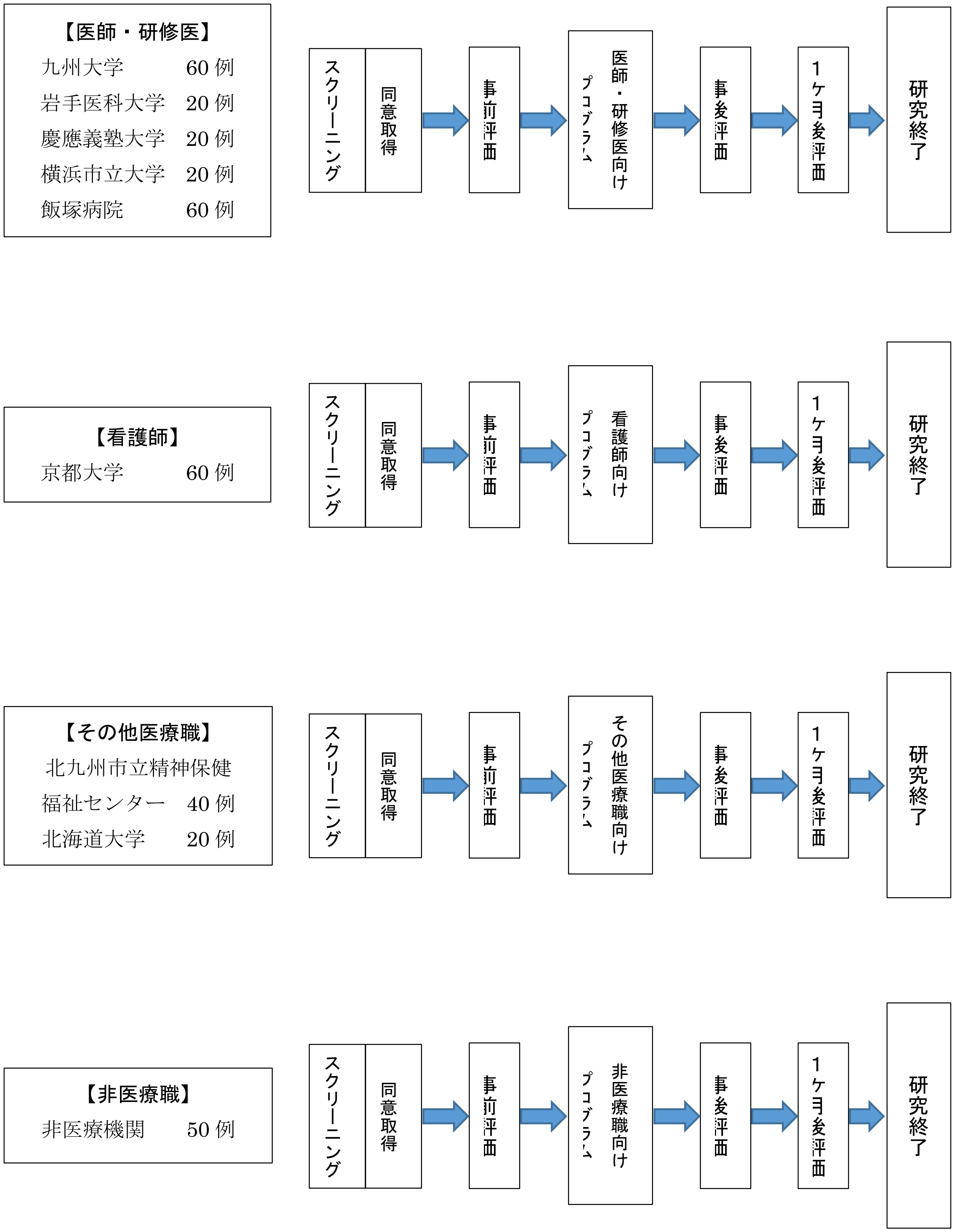
【医師・研修医】
九州大学 60 例
岩手医科大学 20 例
慶應義塾大学 20 例
横浜市立大学 20 例
飯塚病院 60 例
スクリーニング
同意取得
事前評価
医師・研修医向けプログラム
事後評価
1ケ月後評価
研究終了
【看護師】
京都大学 60 例
スクリーニング
同意取得
事前評価
看護師向けプログラム
事後評価
1ケ月後評価
研究終了
【その他医療職】
北九州市立精神保健福祉センター 40 例
北海道大学 20 例
スクリーニング
同意取得
事前評価
その他医療職向けプログラム
事後評価
1ケ月後評価
研究終了
【非医療職】
非医療機関 50 例
スクリーニング
同意取得
事前評価
非医療職向けプログラム
事後評価
1ケ月後評価
研究終了

図 1. 研究の概略

＜研修プログラム＞

MHFA に基づき、我が国の医療現場・医療従事者の実情に則して開発した研修プログラムを用いる。オリジナルの MHFA は 12 時間コースであるが、忙しい医療従事者が大きな負担なく受講できる時間は 2 時間以内であると想定し、プログラムは 2 時間以下としている。プログラムのコンテンツには講義および実習（DVD 教材の視聴とシナリオを用いたロールプレイ）が含まれる。

［講義］

精神疾患に関する知識と、対処スキル向上を図る講義を実施する。

講義には、以下の内容が含まれる

・（講義 1）MHFA って何？
・（講義 2） うつ病と自殺に関する基礎知識
・（講義 3）MHFA の具体的な 5 ステップ（図 2）

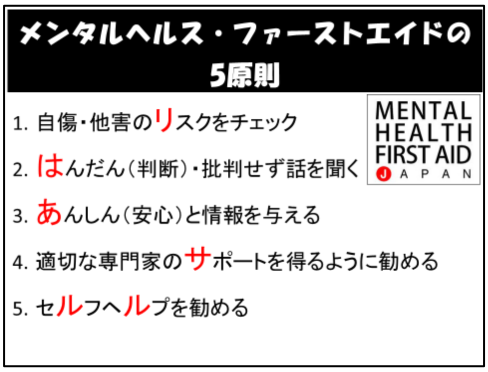

図 2.　講義スライド（抜粋）

［DVD 教材］

実際の医療場面を想定して、MHFA に基づく適切な対応と不適切な対応を収録している。具体的には、抑うつ気分を訴える患者役に対して、医療スタッフ役が対応するというロールプレイに基づき、MHFA の考え方や対応のポイントを解説しており、MHFA に基づく適切な対応・不適切な対応を直感的に把握できるよう配慮されている。DVD 教材は、内閣府ゲートキーパー養成研修プログラムにおける視覚教材を企画・制作してきた大塚（岩手医科大学）を中心に作成された。

［シナリオロールプレイ］

DVD 教材の視聴後、MHFA に基づく具体的な介入法を、DVD 教材のロールプレイを参照して、講義する。その後 3～8 名程度の小グループ（インストラクター1 名に対して参加者 3～8 名）に分かれて、配布したシナリオに沿ってロールプレイを実施するとともに、各インストラクターによるフィードバックの中で、適切な対応法について議論する。最後に、MHFA の要点を簡潔にレビューする。

図 3.　DVD 教材（抜粋）

各プログラムは、受講者に以下の点を重点的に習得できるよう配慮されている。

【医師・研修医向けプログラム】

・精神疾患罹患の可能性を判断できる根拠となる精神医学的知識
・専門科につなぐための具体的なスキル（傾聴、提案のスキル等）

【看護師向けプログラム】

・患者さんの言動から精神疾患罹患の可能性を判断するための知識
・患者さんが安心できる声かけ、話の聴き方に関するスキル

【その他医療職向けプログラム】
・それぞれの職業領域において精神疾患を見逃さないための知識
・患者さんが安心できる情報提供の方法に関するスキル

【非医療職向けプログラム】
・精神疾患が疑われる者を専門家につなぐための具体的な援助行動スキルおよび知識
・精神疾患に対する偏見の低減につながる知識

＜研修プログラム実施＞

研修プログラムを実施し、効果判定を行う。効果判定には、精神疾患に関する知識、対処の自信、自殺念慮への対応の程度を、自記式の調査票を用いて行う。プログラム実施 1 ヶ月後の効果判定においては、参加者の便益を考慮し、調査への回答を自記式調査票またはオンラインの入力フォームから選択できるものとする。

①プログラム実施前評価

研修プログラムの実施前に、参加者へ調査票（プログラムごとに別紙 1、別紙 4、別紙 7、別紙 10）を配布し、回答してもらう。調査票は以下の 1)〜6）から構成される。5）USP scale は医療従事者を想定した尺度であるため、医師・研修医向けプログラム、看護師向けプログラム、その他医療職向けプログラム受講者に対して実施される。非医療職向けプログラム受講者に対しては 5) USP scale の代わりに 6）Link スティグマ尺度が実施される。

1） プログラム参加者の基礎属性：性別、年齢、最終学歴、職種、プログラム参加理由、精神保健上の問題の経験の有無
2） 知識:うつ病ケースの記述を用いた、精神疾患の認識、治療法に関する知識。
3） 自信：精神保健上の問題をもった人に対応する際の自信
4） 行動：精神保健上の問題をもった人に対して実際に提供した支援
5） 自殺企図症例に対する態度尺度：The Understanding Suicidal Patients (USP) scale
6） Link スティグマ尺度（精神科治療に対する差別的態度を測定する尺度）

1)〜6)の調査票は、プログラム実施後評価およびプログラム実施 1 ヶ月後評価において、同一のものが使用される。

②プログラム実施およびプログラム実施後評価

参加者は職種によって、以下 A〜D のプログラムのうちいずれか 1 種類を受講する。

A） 医師・研修医向けプログラム
B） 看護師向けプログラム
C） その他医療職向けプログラム
D） 非医療職向けプログラム

研修プログラム終了直後に、参加者へ調査票（プログラムごとに別紙 2、別紙 5、別紙 8、別紙 11）を配布し、回答してもらう。調査票ではプログラム実施前評価の 1)～6)の項目に加え、プログラムに対する満足度、要望などを尋ねる。また、③プログラム実施 1 ヶ月後評価において、調査票を郵送形式とオンラインの入力フォーム形式のどちらを希望するかを尋ねる。

③プログラム実施 1 ヶ月後評価

研修プログラムの終了から 1 ヶ月後に評価を行う。調査票に関して郵送形式を希望した参加者には、調査票（プログラムごとに別紙 3、別紙 6、別紙 9、別紙 12）を郵送する。オンラインの入力フォームを希望した参加者には、入力フォームの URL をメールにて送信する。調査票はプログラム実施前評価の 1)～6)と同一である。

5. スケジュールあるいは研究期間

承認日　～　平成 31 年 3 月 31 日

6. 予期される利益と不利益

利益：

参加者の精神医学的知識・スキルが向上し、精神疾患患者に対する早期発見および早期介入につながることで、自殺予防をはじめとした患者の福祉の向上に資する。また、精神医学的知識を身につけることで、参加者自身のメンタルヘルスの向上に資することが期待される。

不利益：

介入は最大で 2 時間の講義および実習であり、侵襲性は低いと考えられる。しかし、日常では見慣れない精神疾患に関する講義で、参加者の精神状態が悪化する可能性も完全には否定出来ない。講義の結果として、参加者が自身の精神状態について懸念をいだいた場合は、研究参加中止を含め、研究責任者または研究分担者が適切に対応する。

また、本研究は患者への対応スキルに関する研修プログラムを職種によって 4 種類実施するが、いずれのプログラムも精神疾患患者を早期発見し、精神科や心療内科といった専門科につなぐことを目的としている。したがって、本研修プログラムを受講することにより、患者にとって不適切な対応となることは想定しにくく、患者が被る不利益はないと考えられる。

7. 評価項目

Primary：

・プログラム実施前と実施直後における知識の変化量、および、プログラム実施前と実施 1 ヵ月後における知識の変化量

・プログラム実施前と実施直後における USP scale 得点の変化量、および、プログラム実施前と実施 1 ヵ月後における USP scale 得点の変化量

Secondary：

・プログラム実施前と実施直後における自信の変化量、および、プログラム実施前と実施 1 ヵ月後における自信の変化量

・プログラム実施前と実施直後における Link スティグマ尺度得点の変化量、および、プログラム実施前と実施 1 ヵ月後における Link スティグマ尺度得点の変化量

8. 統計的事項

目標例数：

研修プログラム実施：350 例

データ解析

医師・研修医向けプログラム、看護師向けプログラム、その他医療職向けプログラム、非医療職向けプログラムそれぞれの受講者について、研修プログラム実施前、実施直後、実施 1 ヶ月後それぞれの得点の比較を行う。

9. 倫理的事項：同意書に関すること

本研究は、「ヘルシンキ宣言」および「人を対象とする医学系研究に関する倫理指針（平成 26 年 12 月 22 日改正）」を遵守して実施する。

研究分担者は本研究に先立ち、参加者として適切と思われる者に対し、本研究について別添説明文書を用いて十分な説明をする。その際、質問する機会と研究に参加するか否かを判断するのに十分な時間を与える。説明文書を用いた説明の後、本研究への参加についての判断は参加者本人の自由意思による。参加の有無により参加者の利益又は不利益になるようなことはなく、また、一旦同意した後に、同意を取り消すことも可能であり、同意を取り消したことにより参加者に不利益になることもない旨、参加者に十分説明した上で、同意を取得する。

参加者本人の自由意思による同意が得られたときは、同意書に参加者の署名及び同意日を得る。

10. 健康被害発生時の対処方法

介入は最大 2 時間の講義および実習であり、侵襲性は低いと考えられる。しかし、日常では見慣れない精神疾患に関する講義で、参加者の精神状態が悪化する可能性も完全には否定出来ない。講義の結果として、参加者が自身の精神状態について懸念をいだいた場合は、研究参加中止を含め、研究責任者または研究分担者が適切に対応する。具体的には、研究分担者である精神科医または臨床心理士が参加者の話を傾聴するなど心理的ケアを図り、経過観察を行う。場合によっては、精神科医による治療なども検討する。

また、本研修プログラムを受講することにより、患者に対する受講者の対応が不適切となることは想定しにくく、患者に対して侵襲性はないと考えられることから、健康被害発

生時の対処は特にない。

11. 個人情報の保護方法

参加者の個人情報の取り扱いには十分注意し、参加者個人が特定されることを防ぐため、匿名化を行う。

他施設（岩手医科大学、慶応義塾大学、北海道大学、横浜市立大学、北九州市精神保健福祉センター、飯塚病院、京都大学、非医療機関）にて採取した情報は、共同研究者の責任の下（非医療機関については九州大学の責任の下）、各施設内において匿名化した後、九州大学大学院医学研究院精神病態医学分野に送付する。

参加者が参加を辞退、また同意を撤回した場合は参加者のデータは破棄されるが、その他の場合は保持される。

12. 研究費とその由来

平成 26～29 年度厚生労働科学研究委託費（障害者対策総合研究開発事業(精神障害分野))、研究課題名（公募番号・代表）:精神疾患患者早期介入のための医療従事者向け研修プログラム開発ーメンタルヘルス・ファーストエイドの応用ー(26350901・代表 加藤隆弘)

平成 29～31 年度国立研究開発法人　日本医療開発研究機構（AEMD)（障害者対策総合研究開発事業)、研究課題名:社会的ひきこもりの長期化打開のためのエビデンスに基づく家族向け教育支援モデルの構築（代表　加藤隆弘）

13. 利益相反の有無

本研究の実施に際し、特に問題となる利益相反関係はない。

14. データの二次利用について

本研究において得られたデータ等は、九州大学大学院医学研究院精神病態医学分野において、同分野教授　神庭重信の責任の下、研究期間終了後５年間保存した後、登録番号等を消去し、廃棄する。

上記のデータ等のうち、あらかじめ文書で同意を得られたものについては、将来別の医学研究に二次利用する目的で、前述の保存期間を超えて保存する。二次利用するデータ等は将来新たに計画・実施される医学研究が倫理審査委員会で承認された後に利用する。

15. 研究組織

研究責任者：九州大学大学院医学研究院精神病態医学分野・教授・神庭　重信

研究分担者：九州大学大学院医学研究院精神病態医学分野・特任准教授・加藤　隆弘
（事務局、及び、研究計画書作成担当者）

九州大学病院臨床教育研修センター・センター長/九州大学大学院医学研究院病態修復内科学分野・教授・赤司浩一

九州大学大学院医学研究院精神病態医学分野・ﾃｸﾆｶﾙｽﾀｯﾌ・久保　浩明

九州大学大学院医学研究院精神病態医学分野・ﾃｸﾆｶﾙｽﾀｯﾌ・香月　亮子

九州大学大学院医学系学府精神病態医学分野・共同研究員・早川　宏平

九州大学大学院医学系学府精神病態医学分野・共同研究員・佐藤　美那

九州大学大学院医学系学府精神病態医学分野・大学院生・下川　憲宏

九州大学大学院医学系学府精神病態医学分野・大学院生・金子　祥恵

九州大学大学院医学系学府精神病態医学分野・大学院生・桑野　信貴

共同研究者：岩手医科大学医学部神経精神科学講座・教授・大塚　耕太郎

国立精神・神経医療研究センター　精神保健研究所成人精神保健研究部・室長・鈴木　友理子

慶應義塾大学医学部精神・神経科・専任講師・藤澤　大介

北海道大学大学院医学研究科・客員研究員・橋本　直樹

横浜市立大学附属病院児童精神科・助教・青山　久美

北九州市精神保健福祉センター・所長・三井　敏子

飯塚病院総合診療科・医師・小田　浩之

京都大学医学部附属病院精神科神経科・院内講師・杉原　玄一

京都大学医学部附属病院精神科神経科・大学院生・中神　由香子

研究事務局：

九州大学病院・精神神経科・気分障害外来

連絡先担当者：加藤　隆弘

電話：092-642-####（平日 8:30～17:15）

092-642-####（夜間・休日）

E-mail：takahiro@npsych.med.kyushu-u.ac.jp

16. 研究成果の発表方法

本研究の成果は国際・国内学会発表及び論文発表を予定している。その際、個人を識別できる情報は一切含まない。

17. その他

特記事項なし。
